# 肿瘤免疫疗法中细胞来源对治疗效果的影响

**DOI:** 10.3760/cma.j.cn121090-20240321-00104

**Published:** 2024-07

**Authors:** 文昕 齐, 伟龙 张, 红梅 景

**Affiliations:** 北京大学第三医院血液科，北京 100191 Department of Hematology, Peking University Third Hospital, Beijing 100191, China

## Abstract

从免疫细胞层面总结了各种新型嵌合抗原受体T细胞（CAR-T细胞）疗法，健康年轻受试者的T细胞、干性记忆样T细胞、人体诱导多能干细胞、脐带血T细胞来源的CAR-T细胞疗法可增强肿瘤杀伤效应。从通用型CAR细胞方面，病毒特异性T细胞、γδT细胞、iNKT细胞、巨噬细胞等能有效减轻移植物抗宿主病反应。此外，增强去白细胞过程中单核细胞的清除、保持CD4^+^/CD8^+^ T细胞均衡比例等策略也是增强CAR-T细胞扩增及杀伤效力的方法。细胞耗竭标志物高表达的T细胞对CAR-T细胞的转导、扩增及发挥杀伤效力等会产生负向影响，使用免疫检查点阻断剂等方式抑制T细胞耗竭能提高CAR-T细胞作用效应。

## Abstract (en)

Here we summarized novel Chimeric antigen receptor T-cell immunotherapy（CAR-T）based on the immune material aspect. Young healthy donor T cells, stem cell-like memory T cells, human induced pluripotent stem cells and umbilical cord blood T cells are all potential candidates to enhance CAR-T cell therapy depending on their anti-tumor efficacy. Besides, due to less restricted major histocompatibility complex（MHC）mismatch effect, viral specific T cells, γδT cells, invariant natural killer T cells and macrophages also become idealized T cell sources in terms of Universal CAR-T（UCAR-T）cell therapeutics. In addition, studies demonstrated that more balanced CD4^+^/CD8^+^ T cell ratio and eliminating monocytes during leukapheresis have a positive influence on CAR-T cell functioning, whereas T cells with higher exhaustion markers expression hampers anti-tumor ability of CAR-T cells after infusion. To avoid application of such T cells or mitigate the impact using immune checkpoint inhibitors is of great importance.

作为血液系统肿瘤以及其他实体瘤治疗的新兴策略，嵌合抗原受体T细胞（CAR-T细胞）疗法广泛应用于临床。然而，复发难治性急性淋巴细胞白血病或其他血液系统肿瘤（如慢性淋巴细胞白血病、弥漫大B细胞淋巴瘤、套细胞淋巴瘤、多发性骨髓瘤等）需接受高强度化疗的患者，其T细胞多数量缺乏或功能低下，CAR-T细胞的收集及回输的疗效受极大影响。并且，由于CAR-T细胞制作周期偏长，大多数患者常需要桥接治疗，一些疾病进展快速的患者在接受治疗之前可能面临死亡的风险。为获得在临床上更有效和低毒性的CAR-T细胞，科学家们进行了多靶点CAR-T细胞开发、同种异体CAR-T细胞（UCAR-T细胞）研究等尝试。UCAR-T细胞是从健康供者体内采集的T细胞，并在体外通过基因工程技术改造，制备成现货型的细胞药物[1]。与常规CAR-T细胞相比，UCAR-T细胞不受患者自身T细胞数量和质量影响，其制备安全性和成功率较高，且批量生产可使更多的肿瘤患者受益。除了从健康供者体内获取T细胞，经多能干细胞基因工程方法获得CAR-T细胞、CAR-NK细胞、CAR-巨噬细胞等的研究也日益增多。免疫细胞获取是制备CAR细胞的重要环节之一。不同免疫细胞来源的CAR可能对肿瘤杀灭效果产生正面或负面的影响。从免疫细胞类型的方向优化CAR-T细胞疗法，目前尚无系统性总结。本文综述目前国内外CAR免疫细胞治疗在恶性肿瘤领域的文献，了解新型CAR-T细胞或其他CAR功能细胞的研究进展。

一、T细胞来源及影响因素（图1）

1. 年轻健康人群（Healthy Donor）的T细胞：在UCAR-T细胞相关临床试验中，HD的外周血T细胞是常被采用的对象。一项研究比较了HD和T细胞受体敲除后HD（HD TCR^KO^）CAR-T细胞与B细胞淋巴瘤患者CAR-T细胞的功能。结果显示，HD（13例）和HD TCR^KO^（10例）CAR-T细胞的幼稚和中央记忆CAR-T细胞比例均高于来自淋巴瘤患者的CAR-T细胞（6例）。并且，前者表达了更高比例的CD8^+^CD27^+^PD-1^−^ CAR-T细胞[2]。同样的，一项同种异体CD33靶向CAR-T细胞治疗急性髓系白血病（AML）的研究显示，年轻HD（年龄<30岁）的T细胞经基因编辑技术可改造成以CD33为靶向的HLA-Ⅰ^KO^/TCR^KO^ CD33 UCAR-T细胞，与AML患者自身CAR-T细胞中高表达的耗竭标志物（PD-1及LAG3）等相比，年轻HD中有丰富的记忆表型细胞，其抗肿瘤效应也较后者升高[3]。这些CD33 UCAR-T细胞或可成为治疗AML的潜在选择。

**图1:**
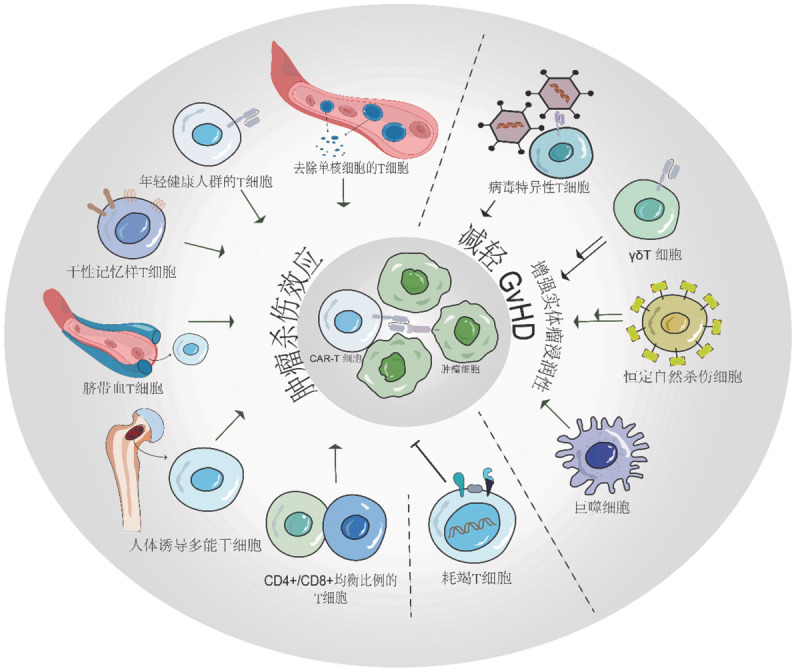
嵌合抗原受体T细胞（CAR-T）免疫疗法细胞来源 **注** 健康年轻受试者的T细胞、干性记忆样T细胞、人体诱导多能干细胞、脐带血T细胞均能增强CAR-T细胞肿瘤杀伤能力。增强去白细胞过程中增强单核细胞的清除、保持CD4^+^/CD8^+^ T细胞均衡比例也是增强CAR-T细胞扩增及杀伤效力的方法。耗竭T细胞会抑制CAR-T细胞的抗肿瘤效应。病毒特异性T细胞可以减轻移植物抗宿主病（GVHD）反应；γδT细胞、恒定自然杀伤细胞既能减轻GVHD反应，还能增强在实体瘤中的浸润。巨噬细胞可以实现良好的实体瘤浸润作用

2. 干性记忆样T细胞（T-SCM细胞）：T-SCM细胞是幼稚T细胞经过抗原激活后，产生的具有长期记忆性的，并能够归巢到淋巴结接受抗原再刺激的T细胞，与普通T细胞相比，中央记忆细胞具有长效的稳定性，更强的扩增能力和肿瘤杀伤能力[4]。T-SCM细胞是记忆T细胞种群中分化程度最低的细胞。其表面同时表达了幼稚T细胞（CCR7^+^、CD45RA^+^、CD45RO^−^、CD27^+^、CD62L^+^）与记忆T细胞［CD95^+^、IL-2Rβ^+^（CD122）、CXCR3^+^、LFA-1^+^］相关的标志物。与成熟T细胞相比，T-SCM细胞衰亡基因如颗粒酶A，杀伤细胞凝集素样受体G1等的表达更低，这些优势使T-SCM细胞比分化后期的T细胞具备更强的分裂扩增能力及肿瘤杀伤能力[4]。最近，一个研究团队发现，再次暴露于肿瘤抗原后，健康供者体内的CD8^+^ T-SCM细胞CD122或CXCR3的表面表达水平越高，其在增殖、自我更新和多能性以及三种细胞因子（TNF-α、IL-2、IFN-γ）的合成释放方面在中都表现出更强的优越性（尤其是CD122）[5]。CD30 CAR-T细胞是霍奇金淋巴瘤一个治疗靶点，既往有研究发现CD30 CAR-T细胞可以被CD3/CD28双表达分子激活。此外，在其体外培养过程中联合细胞因子IL-7、IL-15和IL-21可以促进T-SCM细胞的增殖。并且，他们发现小鼠体内的CD30-CAR-T-SCM细胞的占T细胞比例高者（≥50％）比占比低者（<30％）具有更强的持久性以及杀灭肿瘤细胞的能力[6]。另一项研究在对比了58例复发/难治性弥漫大B细胞淋巴瘤患者接受CAR-T治疗后缓解情况时发现，CD8^+^ T-SCM在CAR-T细胞中含量>15％的患者的临床获益与长期无病生存率显著优于其含量<15％的患者[7]。由此可见，T-SCM细胞在CAR-T细胞制备中具有较强的生存优势。由于外周血中T-SCM细胞的占比较低（占CD4^+^/CD8^+^ T细胞总数的2％～4％），如何诱导足够多的T-SCM细胞以达到过继性免疫治疗所需的细胞水平是一个亟需解决的问题。一些临床前试验发现细胞因子IL-5、IL-17对T-SCM细胞的诱导起促进作用。此外，通过调控信号通路及肿瘤微环境的改变也可影响T-SCM细胞的诱导过程[8]。更多T-SCM参与CAR-T细胞药物合成的相关研究正在进行中，未来T-SCM的应用有望优化CAR-T细胞疗法。

3. 细胞耗竭标志物高表达的T细胞：PD-1、TIM3或LAG3都是细胞耗竭相关因子。细胞耗竭标志物高表达的T细胞影响着CAR-T细胞在患者体内的扩增、转导和杀灭肿瘤细胞的能力。一项调查显示：慢性淋巴细胞性白血病患者接受tisa-cel回输后，无治疗效果患者的T细胞同获得完全缓解患者的T细胞对比，有T细胞耗竭信号通路、效应细胞分化、糖酵解、以及细胞凋亡通路的上调[9]。细胞耗竭标志物通常在细胞活化，高度糖酵解高乳酸环境内高表达。如何避免T细胞耗竭或“重振”耗竭T细胞，使其呈现出“干细胞样”状态可能是进一步突破免疫疗法治疗效果不理想困境的关键。免疫检查点阻断剂（ICB）是实现这一目的最有希望的策略之一。如联合ICB药物或采用基因编辑技术使CAR-T细胞分泌抗PD-1等抗体[25]。并且，有报道证实敲除CAR-T细胞内DNA甲基转移酶3α可以阻止T细胞耗竭并增强及抗肿瘤效应[10]。此外，CD8^+^ T耗竭细胞的特殊子集被称为前体耗竭T细胞（T-_PEX_），最近被发现具有耗竭（PD-1^+^）和记忆（TCF-1^+^、CXCR5^+^）T细胞的特征[11]。T-PEX细胞可能是提示预后的生物标志物，其更新频次是评估细胞耗竭状态的重要参数[11]。在制备CAR-T细胞时联合免疫检查点阻断剂等的使用或可减轻T细胞耗竭。

4. 病毒特异性T细胞（VST）：移植免疫排斥反应是阻碍UCAR-T发展的主要挑战。供者T细胞抗原受体（TCR）识别患者细胞表面人类白细胞抗原（HLA），可引发移植物抗宿主病（GVHD），造成机体器官损伤。VST细胞的过继转移可以重建抗病毒免疫。EBV病毒T细胞、巨细胞病毒、水痘-带状疱疹病毒、流感病毒等都是常见的材料。VST不仅可以预防免疫缺陷的患者移植后机会性感染，还能降低GVHD的发生率。尽管完整的机制尚不清楚，但VST本质属于记忆型T细胞，其TCR多样性降低可能是降低GVHD风险的原因[12]–[13]。随着技术手段的不断更新发展，目前研究者已可以从血清阳性供者、健康供者外周血或脐带血中成功分离并生产获得大量可用的VST细胞。以CD30（霍奇金淋巴瘤）、HER2（胶质母细胞瘤）和GD2（神经母细胞瘤、骨肉瘤）为靶点的自体CAR-VST细胞已成功制造并输注入患者体内，呈现出的安全性和有效性，证明了VST细胞作为CAR-T细胞治疗的可行性[14]。

5. 人体诱导多能干细胞（iPSC）：无论是自体还是同种异体CAR细胞疗法都面临回输后免疫细胞扩增受限及免疫细胞耗竭的问题。iPSC与CAR技术的结合，是CAR-T细胞研究是新方向。iPSC细胞具有无限繁殖的能力，与肿瘤靶点CAR结合后可保持其扩增能力，并且拥有分化多能性的优点[15]–[16]。有研究发现，尽管T-iPSC细胞有表达内源性αβTCR，但其具备γδT细胞样特征，不受HLA配型限制，具备低移植物抗宿主反应性和浸润实体瘤的能力[15]，它们的抗肿瘤活性已在多种临床环境中得到证实，尤其是血液恶性肿瘤。研究表明，与传统CD19 CAR-T细胞相比，来自相同供者的iPSC CD19 CAR-T细胞中Delta-like 1肽表达的上调可能有助于CD8和Th1细胞的发育以及细胞因子如IFNγ/IL2/IL17/IL4等的释放，从而在小鼠体内表现出较强的肿瘤杀伤效应。此外，iPSC CD19 CAR-T细胞可保持源自初始克隆的TCR的同质表达[16]。然而，iPSC细胞的制备及如何利用基因编辑技术诱导其分化成iPSC CAR-T细胞的难度较高，严重限制了其发展及临床应用。

6. 脐带血T细胞：与成人外周T细胞相比，脐带血T细胞具有快速扩增、移植物抗宿主病患病率较低以及移植物抗肿瘤功效较高等特点。脐带血中含有丰富的造血干细胞、祖细胞，以及幼稚T细胞。脐带血T细胞中会自发表达端粒酶，以实现增殖而不缩短端粒，从而有助于延长T细胞后代的寿命[17]。此外，脐带血T细胞对HLA不匹配的耐受性更强。研究显示，尽管HLA存在差异，但一两个等位基因不匹配的急性白血病儿童接受脐带血T细胞移植后的结果与完全匹配的同种异基因供者移植效果相当[18]。脐带血CD4^+^ T细胞中NFAT相关基因以及细胞因子和趋化因子的整体表达减少可能是脐带血T细胞移植后GVHD反应降低的原因[18]。NFAT是控制异基因移植反应的转录因子，NFAT（活化T细胞核因子）转录因子家族包含5种不同的NFAT蛋白：NFATc1（NFAT2或NFATc）、NFATc2（NFAT1或NFATp）、NFATc3（NFAT4或 NFATx）、NFATc4（NFAT3）和NFAT5。T细胞激活后细胞内钙含量增加，导致去磷酸化，从而激活NFAT蛋白，随后NFAT蛋白从细胞质易位到细胞核，并转录激活参与细胞发育、激活和分化的基因。通过上调细胞因子如IL-3、TNF-α的合成与释放，引起排异反应[18]。一项研究发现脐带血CD4^+^ T细胞与外周血CD4^+^ T细胞在初次刺激后0、6、16 h，NFAT相关基因在脐带血CD4^+^ T细胞中表达更低，包括：TNF-α、IFN-γ、粒细胞巨噬细胞集落刺激因子（GM-CSF）、IL-3、IL-4、IL-5、IL-13、IL-2Rα、CD25、CD40L和巨噬细胞炎症蛋白1α（MIP-1α）。NFAT相关通路在脐带血CD4^+^ T细胞中的下调可能是减轻GVHD的原因[19]。与R/R ALL患者来源的CAR-T细胞相比，脐带血来源的CAR-T细胞在白血病小鼠模型中具有更高的初始T和TCM细胞比例，以及更出色的抗肿瘤效应[20]。

7. 去除单核细胞的T细胞：目前，绝大多数CAR-T细胞的生产依赖于直接从患者身上收集起始材料。起始材料通常通过白细胞去除术获得，收集富含白细胞的血液成分。通过提供触发T细胞分裂的信号来激活T细胞[21]。白细胞分离术产品的成分和质量因捐赠者的不同而存在很大差异，特别是对于患有难治性癌症的个体。尽管使用患者的白细胞分离术可以降低排斥反应的风险，但它会引入起始材料成分的变化以及细胞群的存在，这可能会对CAR-T细胞的活化产生负面影响。T细胞活化需受到CD3/CD28单抗偶联磁珠刺激，单核细胞可吞噬CD3/CD28单抗偶联磁珠，从而影响T细胞活化，继而影响T细胞的扩增和效应。对所得CD3 CAR-T细胞的细胞毒性分析表明，在动力学和靶细胞群减少方面，由更纯的起始材料产生的细胞优于由未分选的白细胞分离术制成的细胞。在基因层面，由分离的CD3^+^ T细胞产生的CAR-T细胞被发现有139个基因，较未分选的白细胞分离术产生的细胞基因更多，且这些基因显著上调/下调，这可能是引起两者治疗效果差异的原因[22]。

8. CD4^+^/CD8^+^均衡比例的T细胞：既往研究表明，人类CD4^+^和CD8^+^ T细胞包含功能和转录上不同的子集，它们在体外扩增和过继转移后在体内增殖和持续存在的能力不同。由纯化的CD8^+^或CD4^+^中央记忆T细胞或初始T细胞在CD19 CAR-T细胞疗法中较异质性T细胞来源的CAR-T产品能发挥更强的肿瘤杀伤能力[6]。通过输注一定比例的源自CD8^+^ T细胞和CD4^+^ T细胞的CD19 CAR-T细胞，可以实现效力的协同增强[23]。另一研究表明，以1∶1比例配制CD4^+^和CD8^+^亚群的CAR-T细胞可增强其输注体内后的抗肿瘤活性[24]。当前CD19 CAR-T细胞试验中使用的输注产品常偏向于以CD4^+^或CD8^+^为主的群体。尽管原因尚不完全清楚，但转导和体外扩增方案的差异，包括T细胞刺激方法、细胞因子、饲养细胞、病毒载体和扩增长度等因素，都可能导致CD4^+^/CD8^+^比例不一致。患者性别、年龄、种族或既往接触史也可能影响CD4^+^/CD8^+^ T细胞比例。当在UCAR-T细胞的背景下，生产更一致的产品变得可能。如一项CAR-T试验尝试将CD4^+^和特定CD8^+^亚群分别纯化和转导，然后以1∶1的比例配制，试验显示出显著的临床反应[6]。一项黑色素瘤CAR-T细胞疗法试验中IL-7培养后有效生成CAR-T细胞。与供者活化细胞中观察到的低CD4^+^/CD8^+^比例相比，IL-7培养后转导的细胞保持了相对平衡的CD4^+^/CD8^+^比例[25]。这也不失为一种较为简单可实现CD4^+^/CD8^+^平衡比例的策略。

二、其他细胞来源

1. 恒定自然杀伤T（iNKT）细胞：iNKT是T细胞的一个特殊亚群，可在先天性免疫和适应性免疫之间交叉出现。其受限的TCR只能识别CD1d呈递的脂质抗原。CD1d是一种在B细胞、抗原呈递细胞和一些上皮组织上表达的非多态性糖脂HLAⅠ样分子，不依赖于传统的HLA识别模式，因而回输患者体内GvHD风险低，是制备UCAR-T细胞的潜在材料，其临床安全性已在异体肿瘤的治疗中被多次证实[26]。从人体出生到成年的整个生命周期中，iNKT细胞数量保持稳定，但个体之间差异很大。它们在所有T细胞中所占比例相对较小，在脐带血和外周血中平均为0.1％～0.2％[26]。在小鼠造血干细胞移植的模型中，iNKT细胞的过继转移可以预防GVHD，同时通过抑制同种反应性T细胞扩增和激活以及诱导供体调节T细胞扩增来保留移植物抗白血病效应（GVL）。一项实验发现，应用CD19 CAR-iNKT细胞治疗淋巴瘤小鼠的抑瘤效果比常规CD19 CAR-T细胞更佳。可能是因为前者除了识别淋巴瘤CD19靶点外还能识别其CD1d靶点，能发挥类似双靶点CAR-T细胞疗法的功效[27]。CD19 CAR-iNKT细胞更快的体内抗淋巴瘤活性为无瘤生存和总体生存的改善奠定了基础。然而，外周血中iNKT细胞的稀缺性阻碍了它们的临床应用。一项由脐带血造血干细胞改造的iNKT细胞的早期研究显示，其在改善GVHD同时保留GVL方面具有良好的临床前结果，增强体外扩增或由脐带血细胞中获取或是发展CAR-iNKT细胞的重要来源。

2. γδT细胞：虽然γδT细胞只占循环T淋巴细胞的1％～5％，但它们在上皮组织如皮肤、生殖器官、小肠等中有显著的表达。γδT细胞的分布特点决定了它在实体肿瘤浸润方面的重要作用[6]。一项研究表明，以叶酸受体α（Fra）为靶点，联合IL-7和CCL19配体的CAR-γδT细胞疗法对患三阴性乳腺癌的小鼠能发挥较强的肿瘤杀伤效应[28]。与αβT细胞通过抗原特异性TCR信号传导激活细胞毒性不同，γδT细胞可以通过细胞表面受体直接诱导细胞毒性，包括γδT细胞受体（TCRγδ）和自然杀伤群2D（NKG2D）受体，一旦激活，这些细胞会释放大量INF-α、IL-17等细胞因子消灭肿瘤细胞。除了降低GVHD的优势，γδT细胞对丢失靶点的肿瘤细胞也能发挥杀伤作用，有望解决当前血液肿瘤中CD19靶抗原丢失或调节导致CAR细胞治疗后耐药和复发的问题[28]。研究发现，在小鼠模型内，CAR-γδT细胞能够靶向攻击CD19抗原阴性白血病细胞，并且，这种效应在联合使用唑来膦酸盐后得到增强[29]。一项以γδT细胞为制备原料的针对B细胞血液肿瘤UCAR-T细胞疗法（NCT04735471）的一期临床试验正在进行中，临床前实验显示CD20 CAR-γδT细胞在有B细胞淋巴瘤异种移植物的免疫缺陷小鼠体内呈现抑制肿瘤生长的作用，并且未见GvHD反应[30]。该免疫疗法有望在血液肿瘤患者体内实现相似的效应。

3. 巨噬细胞：巨噬细胞对实体肿瘤有良好的浸润性，是主要的免疫调节因子，并且在肿瘤微环境中含量较高。巨噬细胞可分为M_1_型与M_2_型。M_1_表型在炎症细胞因子中高表达，具有较强的抗微生物和肿瘤活性，而M_2_可以促进组织重塑和肿瘤生长。M_2_巨噬细胞可被诱导分化为M1表型从而发挥抗肿瘤作用[31]。开发用于癌症免疫治疗的CAR巨噬细胞（CAR-M）可以克服CAR-T/NK细胞疗法在实体瘤中的治疗障碍。一项HER2 CAR-M治疗复发/难治性HER2过度表达的肿瘤患者的Ⅰ期临床试验正在进行中。该研究首次招募了18例HER2过表达实体瘤患者，研究腺病毒转导CAR-M在人体中的效果[32]。然而，巨噬细胞本身也存在较大的缺陷。比如，巨噬细胞回输体内后无法增殖，可能需要重复给药以维持足够的CAR巨噬细胞水平以发挥肿瘤杀伤效应。此外，M_1_～M_2_型间的转化问题，CAR-M经病毒转染的过程中可能诱发插入突变，以及如何在肿瘤微环境中优化CAR-M结构、肿瘤浸润和细胞毒性保留等都是CAR-M发展中亟需解决的难题[31]。

三、总结与展望

综合来看，相比于常规CAR-T细胞，新型CAR-T细胞的制备、质控、临床给药方面进行了简化，但同时也带来了新的挑战，如UCAR-T细胞可能引起的移植免疫排斥反应。目前，临床证据显示健康年轻人群的T细胞、T-SCM细胞、病毒特异性T细胞、人体诱导多能干细胞、脐带血T细胞、γδT细胞、iNKT细胞、巨噬细胞等能有效制备UCAR-T细胞或优化CAR-T细胞治疗。此外，增强去白细胞过程中增强单核细胞的清除、保持CD4^+^/CD8^+^ T细胞均衡比例等策略也是增强CAR-T细胞扩增及杀伤效力的方法。细胞耗竭标志物高表达的T细胞对CAR-T细胞的转导、扩增及发挥杀伤效力等会产生负向影响，使用免疫检查点阻断剂等方式抑制T细胞耗竭能提高CAR-T细胞作用效应（表1）。以上免疫细胞来源的UCAR-T或CAR-T产品在多项血液瘤及实体瘤的临床研究中展现出可喜的临床疗效及安全性，新一代安全有效的CAR-T细胞疗法有望实现更多的可及性，改善血液肿瘤以及其他实体瘤的治疗。

**表1:** 细胞免疫治疗细胞来源

CAR免疫细胞来源及影响因素	作用机制	作用效果	靶点	细胞治疗类型
T细胞来源				
年轻健康人群的T细胞	低分化T细胞占比更高	增强T细胞扩增、转导、杀伤能力	BCMA	UCAR-T[3]
干性记忆样T细胞	同时表达记忆T细胞与幼稚T细胞表面标志物衰亡基因低表达	增强T细胞扩增、转导、杀伤能力	CD19、CD30	CAR-T[6]–[7]
病毒特异性T细胞	TCR多样性降低	减轻GVHD	CD19	UCAR-T[13]
人体诱导多能干细胞	具备干细胞无限增殖能力，具备γδT细胞样特征，不受HLA配型限制	增强T细胞功效及减轻GVHD	CD19	UCAR-T[16]
脐带血T细胞	自发表达端粒酶，以实现增殖而不缩短端粒，延长T细胞寿命，与GVHD相关基因NFAT家族及趋化因子等低表达	增强T细胞功效及减轻GVHD	CD19	UCAR-T[18]
保持CD4^+^/CD8^+^ T细胞均衡比例	CD4^+^/CD8^+^ T细胞协同增强作用	增强T细胞扩增、转导、杀伤能力	CD19	CAR-T[25]
清除单核细胞	T细胞活化需受到CD3/CD28微球刺激，单核细胞可吞噬CD3/CD28微球，从而影响T细胞活化	增强T细胞扩增、转导、杀伤能力	CD19	CAR-T[21]
细胞耗竭因子高表达的T细胞	与T细胞耗竭信号通路、效应细胞分化、糖酵解以及细胞凋亡通路的上调相关	阻碍T细胞杀伤能力	CD19	CAR-T[9]
其他细胞来源				
γδT细胞	多分布于上皮组织，γδT受体不受HLA配型限制	减轻GVHD，实体瘤浸润好	CD20	UCAR-γδT[29]
iNKT细胞	其TCR只能识别CD1d呈递的脂质抗原,不依赖于特定的HLA	减轻GVHD	CD19	CAR-iNKT[27]
巨噬细胞	肿瘤微环境中含量较高不受HLA配型限制	减轻GVHD，实体瘤浸润好	HER-2	CAR-M[32]

**注** CAR：嵌合抗原受体；GVHD：移植物抗宿主病；BCMA：B细胞成熟抗原；UCAR-T：异体CAR-T细胞；CAR-M：CAR巨噬细胞
